# Down-Regulation of Myogenin Can Reverse Terminal Muscle Cell Differentiation

**DOI:** 10.1371/journal.pone.0029896

**Published:** 2012-01-03

**Authors:** Nikolaos P. Mastroyiannopoulos, Paschalis Nicolaou, Mustafa Anayasa, James B. Uney, Leonidas A. Phylactou

**Affiliations:** 1 Department of Molecular Genetics, Function and Therapy, The Cyprus Institute of Neurology and Genetics, Nicosia, Cyprus; 2 The Henry Wellcome Laboratories for Integrative Neuroscience and Endocrinology, University of Bristol, Bristol, United Kingdom; Istituto Dermopatico dell'Immacolata, Italy

## Abstract

Certain higher vertebrates developed the ability to reverse muscle cell differentiation (dedifferentiation) as an additional mechanism to regenerate muscle. Mammals, on the other hand, show limited ability to reverse muscle cell differentiation. Myogenic Regulatory Factors (MRFs), MyoD, myogenin, Myf5 and Myf6 are basic-helix-loop-helix (bHLH) transcription factors essential towards the regulation of myogenesis.

Our current interest is to investigate whether down-regulation of MRFs in terminally differentiated mouse myotubes can induce reversal of muscle cell differentiation. Results from this work showed that reduction of myogenin levels in terminally differentiated mouse myotubes can reverse their differentiation state. Down-regulation of myogenin in terminally differentiated mouse myotubes induces cellular cleavage into mononucleated cells and cell cycle re-entry, as shown by re-initiation of DNA synthesis and increased cyclin D1 and cyclin E2 levels. Finally, we provide evidence that down-regulation of myogenin causes cell cycle re-entry (via down-regulation of MyoD) and cellularisation through separate pathways. These data reveal the important role of myogenin in maintaining terminal muscle cell differentiation and point to a novel mechanism by which muscle cells could be re-activated through its down-regulation.

## Introduction

Vertebrates like zebrafish and salamanders can display unique regenerative abilities through dedifferentiation or differentiation of precursor cells [1]. After injury, these vertebrates are able to induce reversal of the differentiation state, which leads to a series of events that aim to generate proliferating regenerative progenitor cells with the ability to restore in a precise way the lost tissue [1], [2], [3]. In addition, recent studies showed that terminally differentiated mammalian muscle cells are capable to reverse their differentiation state. The course of myogenesis is a well characterized example of terminal differentiation. Myoblasts are capable of proliferation and upon demand to form skeletal muscle, they exit the cell cycle and through the activation of muscle-specific transcription factors they fuse into multinucleated terminally differentiated myotubes [4], [5]. Some research groups have attempted to induce dedifferentiation of muscle cells by exogenous genes or chemicals. Mouse C2C12 myotubes treated with limb regeneration extracts were able to induce myotubes to reenter the cell cycle, exhibited reduced levels of muscle differentiation proteins and cleaved to produce smaller myotubes or proliferating mononucleated cells [6]. In another study, combination of growth medium and ectopic msx1 expression caused the reduction of muscle-specific proteins and the cleavage of these myotubes into proliferating mononucleated cells that were able to redifferentiate into muscle or trans-differentiate into various cell types [7]. In a similar way, overexpression of Twist, a nuclear basic helix-loop-helix (bHLH) transcription factor known to inhibit muscle cell differentiation, in terminally differentiated myotubes caused their cleavage to mononucleated cells and re-entry to the cell cycle [8]. Moreover, microinjection of Barx2 cDNA into immature myotubes derived from primary cells led to cleavage and formation of mononucleated cells that were able to proliferate [9]. Using a chemical approach, terminal differentiated myotubes were incubated with a triazine compound. Myotubes were cellularized into smaller myotubes or mononucleated cells, which were able to survive and divide [10]. Similarly, myoseverin a trisubstituted purine was shown to induce reversible fission of multinucleated myotubes into mononucleated cells, which were able to enter the cell cycle [11]. Recently, mammalian skeletal muscle cells were induced to dedifferentiate into proliferating mononuclear cells, after treatment with myoseverin and temporary p21 suppression. These cells were further induced to act as multipotent stromal cells by further treatment with the small molecule, reversine (2-(4-morpholinoanilino)-6-cyclohexylaminopurine) and simple chemical modifications of the culture media [12]. When cell cycle inhibitors, p21 and p27 were depleted from terminal differentiated mouse myotubes, incomplete DNA replication and apoptosis was observed. In contrast, when p21 and p27 were depleted from quiescent, non-terminal differentiated fibroblasts and muscle cells, DNA replication was fully recovered and apoptosis was no longer observed. These cells were able to proliferate in the absence of growth factors [13]. Recently, evidence for natural dedifferentiation of muscle cells, following injury was reported by using a Cre/Lox-β-galactosidase system [14], [15].

Myogenic regulatory factors (MRFs), myogenin, MyoD, MRF4 (Myf6) and Myf5 are basic-helix-loop-helix (bHLH) transcription factors that regulate myogenesis [16], [17], [18], [19], [20], [21], [22]. MyoD is absent during G0 phase of the cell cycle, but is highly expressed during mid-G1 phase and between S to M phase. Myf5 is highly expressed during G0 and decreases during G1 phase [23]. MyoD was found to promote cell cycle arrest by inducing cyclin-dependent kinase (CDK) inhibitor p21 [24], [25], cyclin D3 [26] and retinoblastoma (Rb) tumor suppression protein [27], [28], all of which have important functions towards cell cycle withdrawal. Interestingly, overexpression of MyoD is able to promote myoblasts to differentiate, while by overexpression of Myf5 fail to differentiate [23]. MyoD is also expressed in myotubes and collaborates with myogenin to regulate the expression of genes necessary for terminal differentiation [29].

Myogenin and Myf6 are expressed upon the differentiation of myoblasts to multinucleated myotubes [22], [30], [31], [32]. Myogenin is essential during differentiation. Mice lacking the myogenin gene die at birth due to severe skeletal muscle deficiency, as myoblasts are unable to fuse into multinucleated myofibers [33]. Furthermore, MyoD and Myf5 are unable to substitute myogenin's functions during differentiation [34]. Mice lacking the myogenin gene express normal levels of MyoD and Myf5 [33].

Here we show that down-regulation of endogenous myogenin gene expression in terminally differentiated mouse muscle cells causes cleavage of myotubes into mononucleated cells and entry to the cell cycle through down-regulation of MyoD, in two different pathways. These results reveal yet another important role for myogenin which is to prevent reversal of muscle cell differentiation.

## Results

### Reduction of terminally differentiated myotubes after down-regulation of endogenous myogenin expression

**Figure 1:**
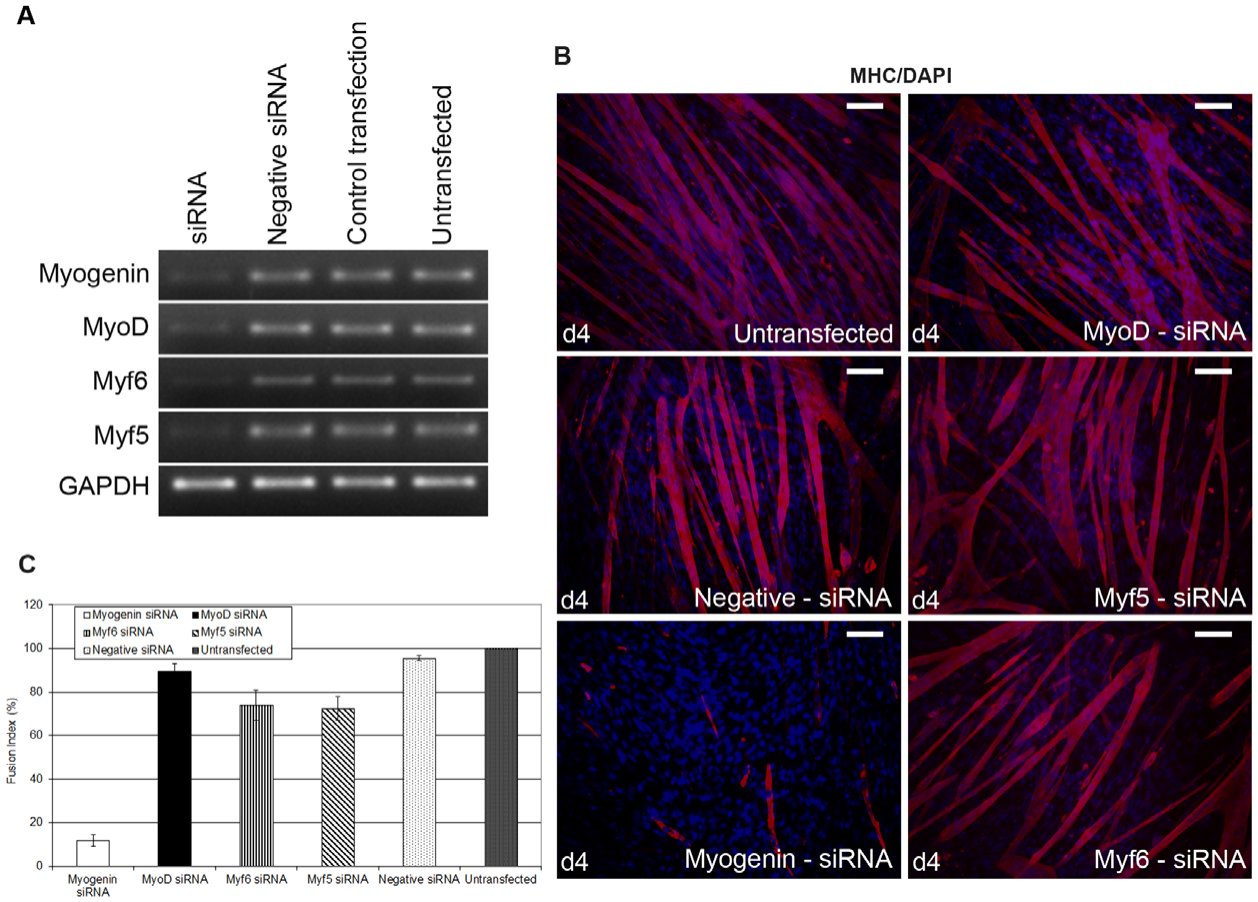
siRNA-mediated down-regulation of myogenin causes reduction of terminally differentiated myotubes. (A) Following differentiation into myotubes, cells were transfected with myogenin, MyoD, Myf6 and Myf5 siRNAs and controls (d0). Growth medium was added to cells two days later (d2) and left for two more days (d4). RNA analysis revealed a substantial reduction at the RNA levels of myogenin, MyoD, Myf6 and Myf5, compared to controls (negative siRNA and control transfection) and untransfected C2C12 cells. GAPDH was used as an internal control. (B) MHC immunostaining revealed a substantial reduction of myotubes in cells transfected with myogenin siRNA compared to cells transfected with MyoD, Myf6, Myf5 and control cells (Scale, 200 µm). (C) Fusion Index was calculated from cells transfected with each siRNA and immunostained with MHC. Fusion Index (FI) was defined as the number of nuclei present in myotubes in comparison over the total number of nuclei present in the observed field. Data was selected from 10 different and randomly chosen microscopic fields. Cells transfected with myogenin siRNA showed significantly lower FI (12%), compared to cells transfected with MyoD (89%), Myf6 (75%), Myf5 (73%), negative siRNA (97%), and untransfected cells set to 100.

As a first step to determine whether MRFs are needed to maintain terminal muscle cell differentiation, terminally differentiated cells were transfected with individual siRNAs, specific for each of the four MRFs (MyoD, Myf5, myogenin, Myf6). C2C12 cells, which are suitable for *in vitro* differentiation were grown to confluency and induced to become multinucleated myotubes, prior to transfections. Following transfections, RNA analysis revealed the expected and specific reduction of mRNA of all MRFs (fig. 1A).

Transfections with individual siRNAs for each of the four MRFs was repeated and left for four days in growth medium in order to determine whether down-regulation of MRFs could have any effect on myotubes. Those cultures which expressed reduced endogenous myogenin had significantly less myotubes (fig. 1B). SiRNA-Myogenin transfected myotubes exhibited a significantly reduced number of myosin heavy chain (MHC) stained myotubes, a terminally differentiation marker, compared to cells, which expressed reduced endogenous levels of MyoD, Myf5 and Myf6 (fig. 1B). Fusion Index (FI), a way to measure muscle cell differentiation was also found to be significantly lower (12%) in cells transfected with myogenin siRNA, compared to cells transfected with MyoD (89%), Myf6 (75%), Myf5 (73%) siRNA, negative siRNA (97%) and untransfected myotubes (100%) (fig. 1C).

### Down-regulation of myogenin in terminally differentiated muscle cells induces myotube cleavage into active mononucleated product cells

**Figure 2:**
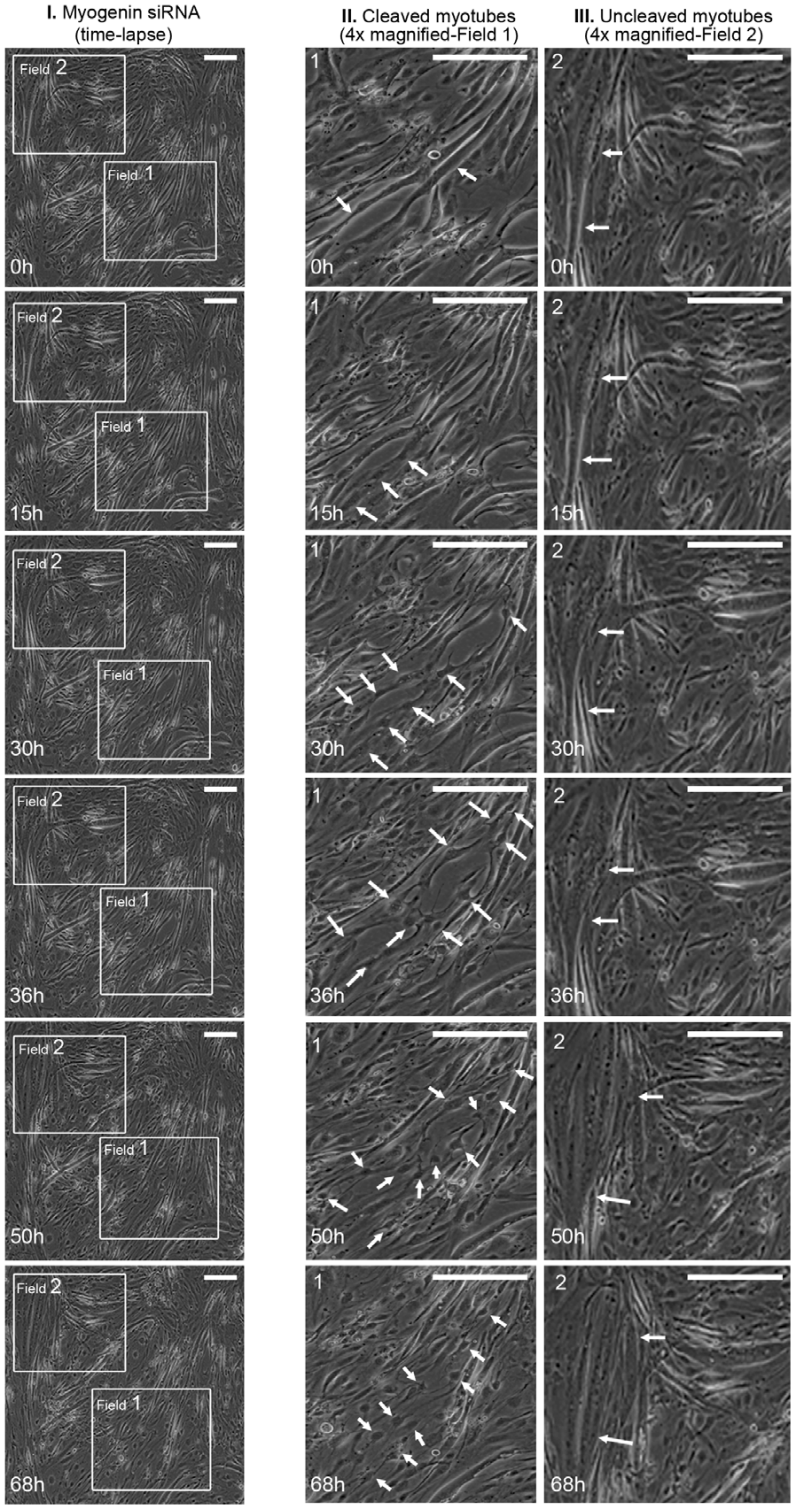
Myogenin siRNA transfection in terminally differentiated myotubes causes their cleavage into active mononucleated cells. Following myotube differentiation, cells were transfected with myogenin siRNA. Growth medium was then added to the cells and placed for time lapse microscopy. (I) (0 h), myotubes just after myogenin siRNA transfection. (0 h–68 h), uncleaved myotube (field 2) and cleaved myotubes (field 1) after myogenin siRNA transfection (Scale, 250 µm). (II) Myotube in field 1 in higher magnification from 0 h–68 h displayed significant morphological changes and cleavage. During 15 h–30 h, myotubes began to morphologically change (arrows indicate the movement of the nuclei and the areas of possible cleavage). During 36 h–68 h, myotubes were completely cleaved into mononucleated cells (arrows indicate cleaved cells) (Scale, 200 µm). (III) Myotube in field 2 in higher magnification from 0 h–68 h showed no signs of cleavage (Scale, 200 µm).

As a result of the large reduction in myotubes, due to down-regulation of endogenous myogenin, experiments were carried out to investigate in more detail the mechanism by which this occurs. The first experiments aimed at looking at the morphological changes which occur in myotubes, following myogenin siRNA transfections. After differentiation of myoblasts into multinucleated myotubes, cells were transfected with myogenin siRNA. Myotubes transfected with myogenin siRNA cleaved into mononucleated cells almost 70 h after transfection, as seen by time-lapse microscopy (fig. 2).

**Figure 3:**
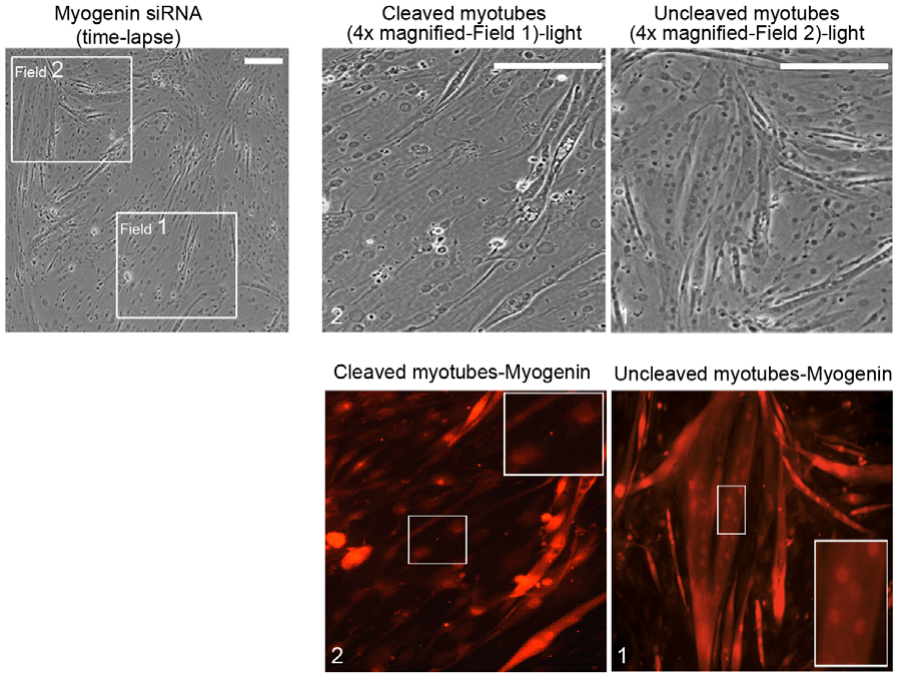
Mononucleated muscle product cells have reduced myogenin levels. Following myogenin siRNA transfection in terminally differentiated myotubes and time lapse microscopy for 68 h (fig. 2), cells were fixed and immunostained with a specific myogenin antibody (Scale, 200 µm). Magnification of the myotube in field 2 showed normal myogenin expression (cleavage was not detected as observed by time lapse in fig. 2). Magnification of the mononucleated product cells in field 1 (after cleavage of myotubes as observed in fig. 2), showed significantly reduced myogenin expression levels (Scale, 200 µm).

This finding indicates that the induced reduction of myogenin endogenous levels in differentiated multinucleated myotubes initiates a cellular mechanism, which results in the cleavage of these cells into mononucleated product cells. Furthermore, product cells obtained from the cleavage of multinucleated myotubes expressed significantly reduced myogenin levels compared to uncleaved myotubes which express normal myogenin levels (fig. 3).

**Figure 4:**
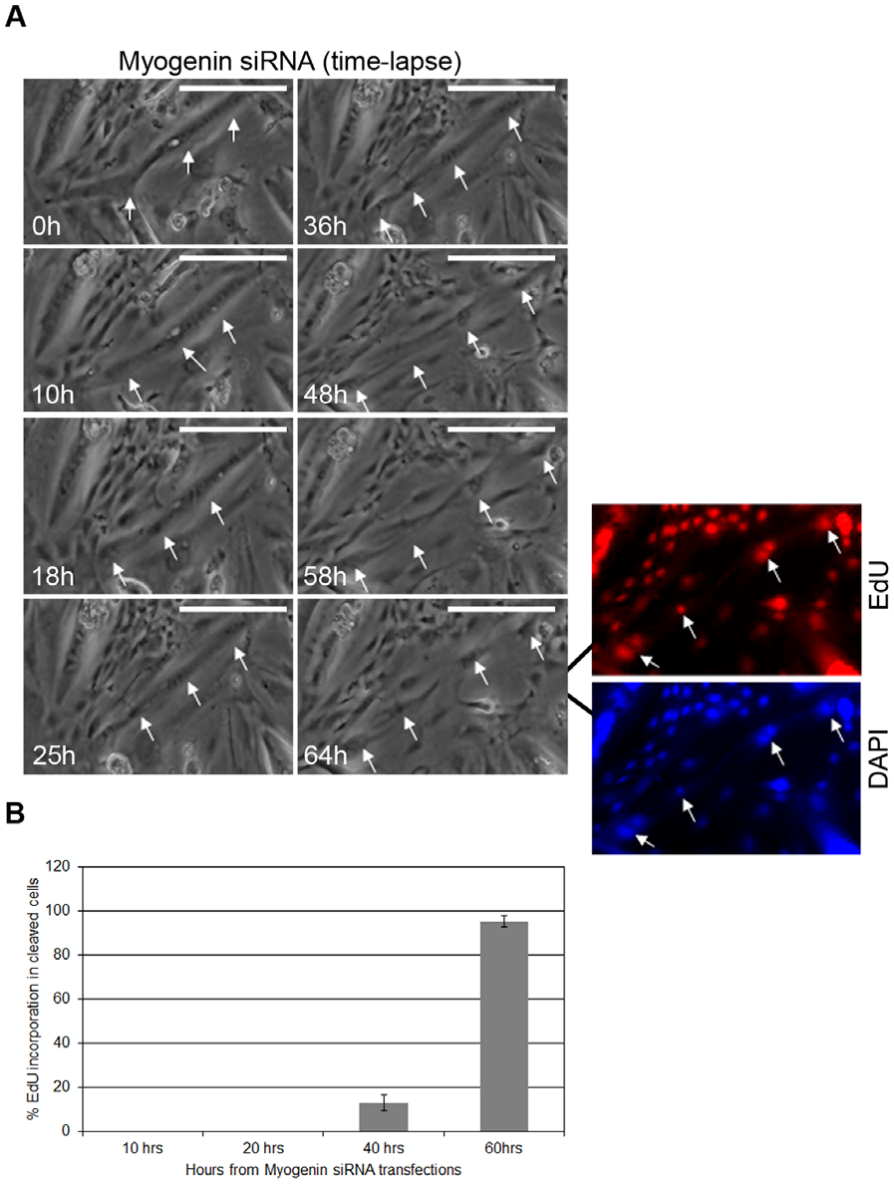
Mononucleated muscle product cells are active in DNA synthesis. (A) Following myotube differentiation, cells were transfected with myogenin siRNA. Growth medium was then added to the cells and placed for time lapse microscopy. 64 h after transfection with myogenin siRNA, myotubes cleaved into mononucleated cells (arrows indicate cleaved cells). Cells were then stained with EdU. Cells which derived from the cleavage of myotubes (indicated by arrows) incorporated high levels of EdU. (B) Following myotube differentiation, cells were transfected with myogenin siRNA. Growth medium was then added to the cells. Following various incubation time points of transfected cells in growth medium, 10, 20, 40 and 60 hours, cells were then stained with EdU. The number of EdU-positive cleaved cells was counted in 5 random microscopic fields for each time point (Scale, 200 µm).

In order to investigate whether product cells, which arise from the down-regulation of endogenous myogenin gene expression, can reenter cell cycle, cells were stained with 5-ethynyl-2′-deoxyuridine (EdU). Cells which derived from the cleavage of myotubes incorporated high levels of EdU (fig. 4), indicating that these cells have active DNA replication.

### Down-regulation of myogenin in terminally differentiated myotubes reactivates cell cycle through MyoD

**Figure 5:**
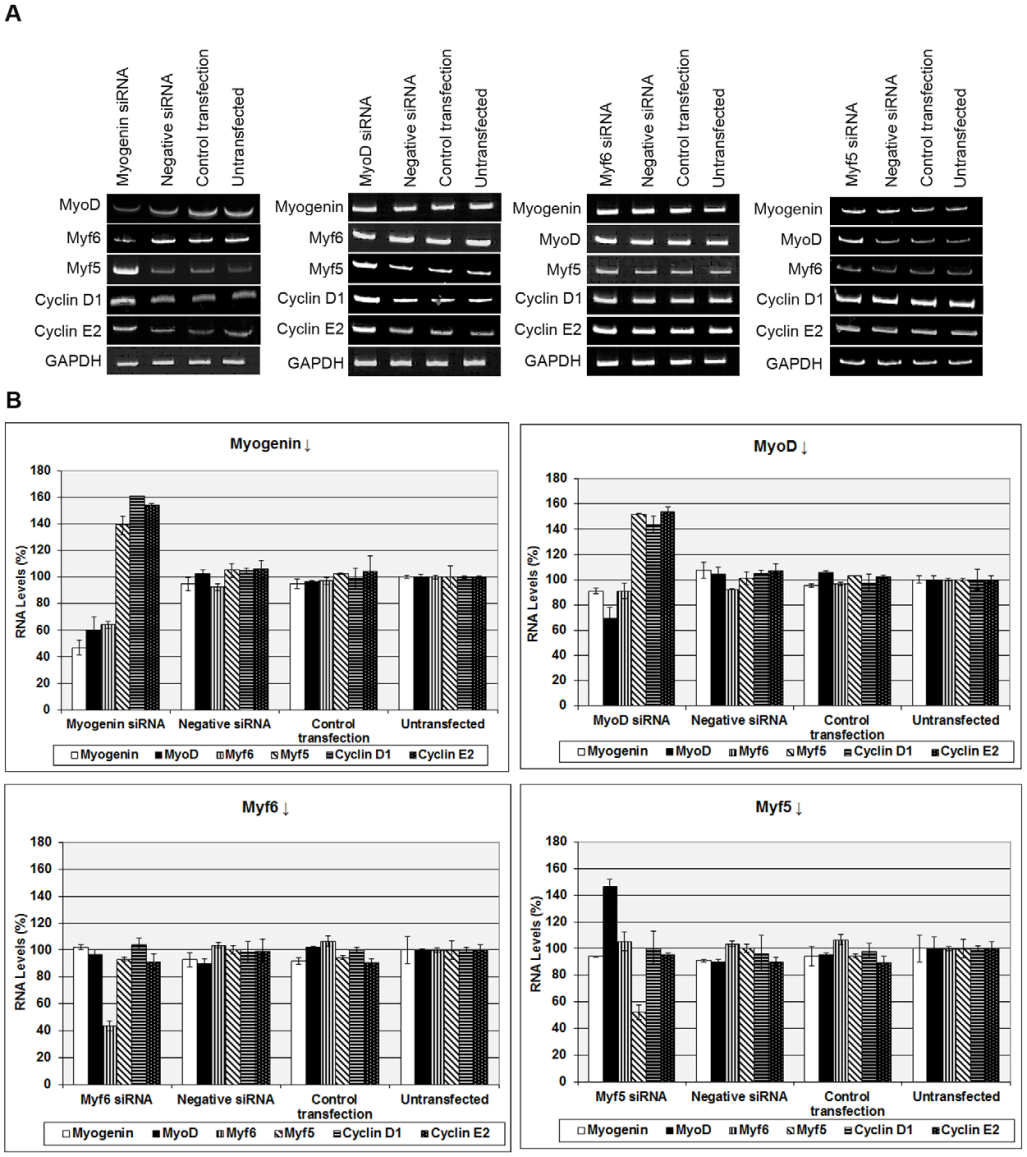
Myogenin and MyoD siRNA transfection in terminally differentiated myotubes reactivates cyclin D1 and cyclin E2. (A) Myoblasts treated with Ara-C and induced to differentiate into multinucleated myotubes were transfected with myogenin, MyoD, Myf6 and Myf5 siRNAs and controls. After transfections, growth medium was added to cells prior to RNA analysis by RT/PCR analysis. Cells transfected with myogenin siRNA revealed reduction at the RNA levels of myogenin, MyoD, Myf6 and increase of cyclinD1, cyclinE2 and Myf5 when compared to controls and untransfected C2C12 cells. Cells transfected with MyoD siRNA revealed reduction of the RNA levels of MyoD and substantial increase of cyclinD1, cyclinE2 and Myf5. Cells transfected with Myf6 siRNA revealed reduction of the RNA levels of Myf6. Cells transfected with Myf5 siRNA revealed reduction of the RNA levels of Myf5 and substantial increase of MyoD. (B) Graphs of the RNA analysis. Values were obtained as ratios of the RNA of interest over GAPDH internal control.

Down-regulation of myogenin caused cleavage of terminally differentiated myotubes into mononucleated product cells, which can reenter into the cell cycle. As a next step, a series of molecular experiments was carried out to reveal changes of important molecules which are implicated in cell cycle activation and differentiation of muscle cells. MyoD levels were lower in cells transfected with myogenin siRNA than control cells, indicating that down-regulation of myogenin may cause the endogenous down-regulation of MyoD (fig. 5A, B). Similarly, Myf6 levels were lower compared to control cells (fig. 5A, B). Interestingly, down-regulation of endogenous myogenin levels caused an increase in Myf5 levels, which may be due to a compensatory effect of the MyoD decrease (fig. 5A, B). Several previous reports showed that MyoD and Myf5 might compensate for each other [35]. Down-regulation of myogenin caused also the induction of cyclins D1 and E2 which are both involved in the G1-S transition of the cell cycle (fig. 5A, B) [36].

SiRNA-mediated down-regulation of MyoD in terminally differentiated myotubes, on the other hand caused similar molecular changes, as those seen in cells transfected with myogenin siRNA. More specifically, reduction of endogenous MyoD levels in terminally differentiated cells resulted in increases of Myf5 (probably to compensate for MyoD reduction), cyclin D1 and E2 levels (fig. 5A, B).

Apart from the increase in MyoD levels, which was caused by the down-regulation of endogenous Myf5, both Myf5 and Myf6 siRNAs had no molecular effects on molecules which are implicated in muscle cell differentiation and the cell cycle.

These molecular results show that down-regulation of myogenin gene expression of terminally differentiated cells alters gene expression which is involved both in muscle cell differentiation and the cell cycle. The decrease in MyoD and the subsequent increases in cyclins D1 and E2 justify the reversal of muscle cell differentiation, as seen by the cleavage of myotubes and the creation of active mononucleated cells. The results also indicate that down-regulation of myogenin leads cells to the cell cycle, probably through the down-regulation of MyoD.

### Cleavage of myotubes and cell cycle reactivation have different pathways

Results so far showed that down-regulation of endogenous myogenin levels caused the cleavage of myotubes into mononucleated cells and lead cells into the cell cycle, probably through down-regulation of MyoD.

**Figure 6:**
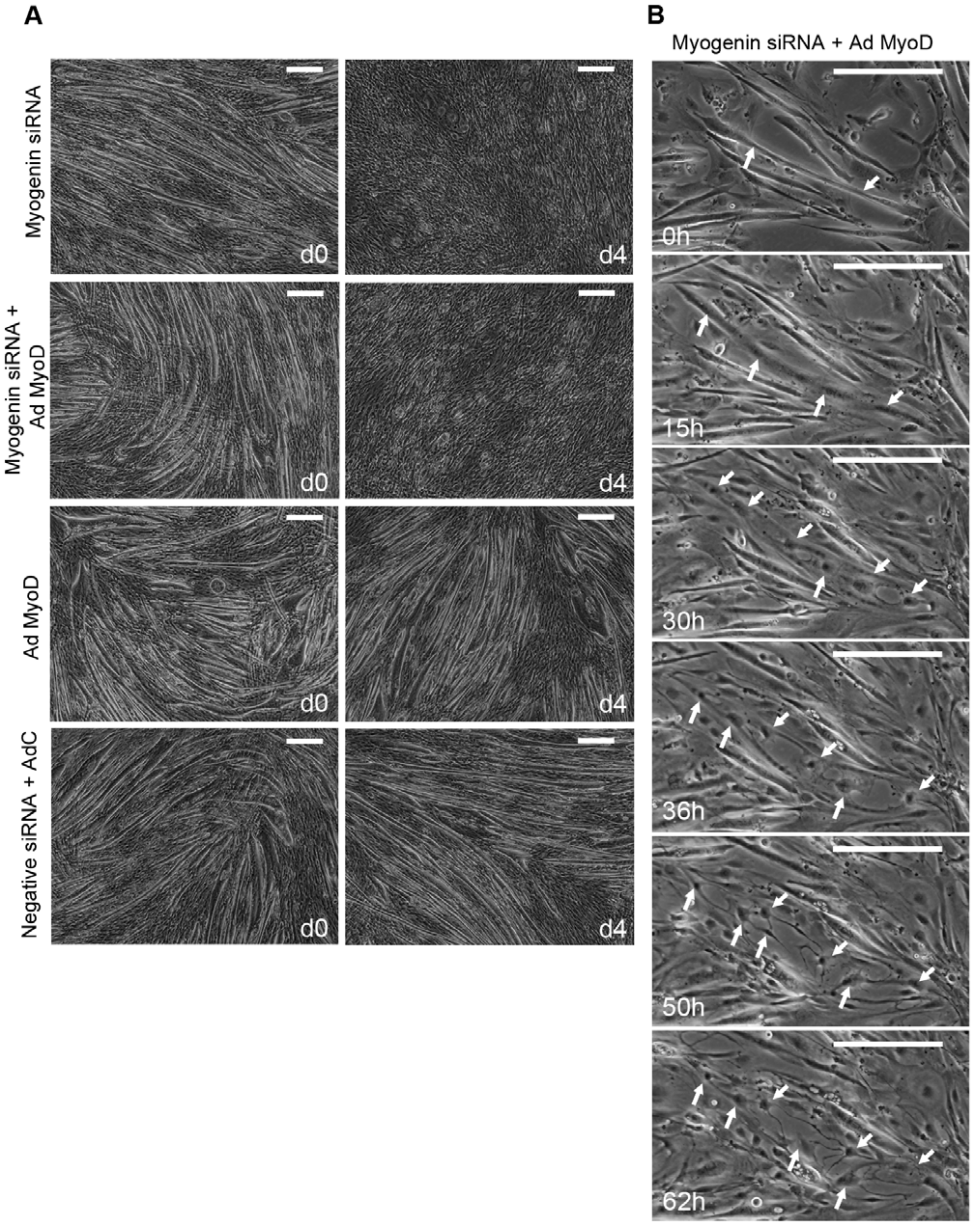
Overexpression of MyoD does not prevent cleavage of siRNA – myogenin myotubes. Myotubes were transfected with myogenin siRNA only, myogenin siRNA co-transfected with an adenovirus expressing MyoD (AdMyoD), AdMyoD only and a negative siRNA co-transfected with a control adenovirus (AdC) (d0). After transfections, growth medium was added to cells (d2) and left for two more days (d4). Cells transfected with myogenin siRNA and myogenin siRNA co-transfected with AdMyoD showed similar reduction of myotubes compared to cells transfected with AdMyoD and negative siRNA co-transfected with AdC (Scale, 200 µm). (B) Time-lapse microscopy of cells co-transfected with myogenin siRNA and AdMyoD showed cleavage of myotubes into mononucleated cells (indicated by arrows) similar to the cells transfected with myogenin siRNA only (fig. 2.) (Scale, 200 µm).

In order to determine the way by which reduction of myogenin drives cells to cleavage and cell cycle, terminally differentiated myotubes were transfected with myogenin siRNA in the presence of an adenovirus, expressing MyoD (AdMyoD). The aim was to prevent cell cycle reactivation by preventing MyoD levels from being reduced through myogenin down-regulation. Overexpression of MyoD and down-regulation of endogenous myogenin levels did not stop myotubes cleavage and cellularisation (fig. 6A, B). No differences in their ability to change morphologically into mononucleated cells were seen compared to myotubes transfected only with myogenin siRNA (fig. 6A).

**Figure 7:**
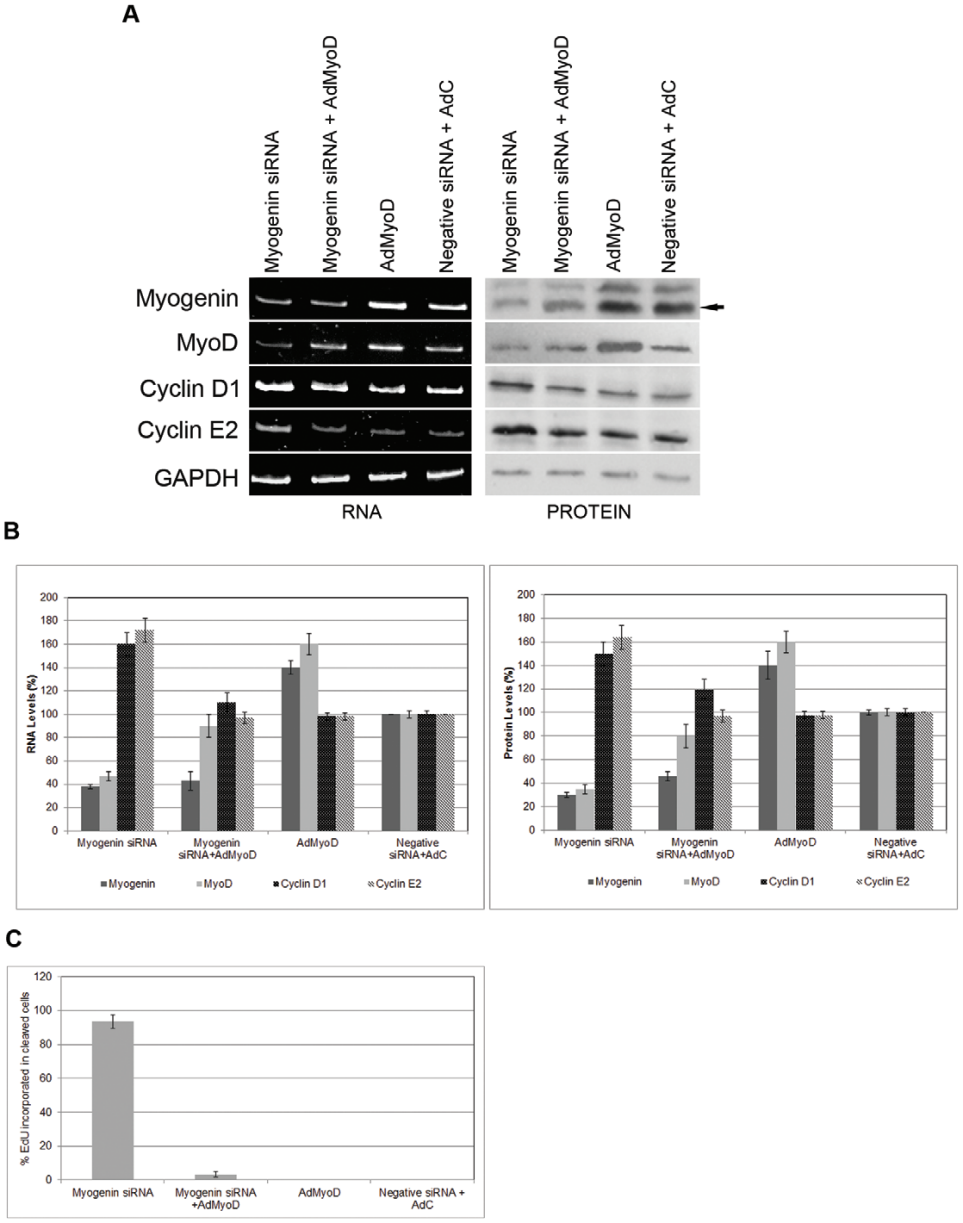
Cleavage of myotubes and cell cycle reactivation have different pathways. (A), (B) RT/PCR and protein analysis on cells co-transfected with myogenin siRNA and AdMyoD showed normal MyoD levels compared to cells transfected with myogenin siRNA only which showed significantly reduced MyoD levels. Cells co-transfected with myogenin siRNA and AdMyoD showed normal cyclin D1 and cyclin E2 levels compared to cells transfected with myogenin siRNA only, which showed to be substantially increased. Cells co-transfected with negative siRNA and AdC revealed similar levels of cyclin D1 and cyclin E2. Cells transfected with AdMyoD showed to some extent higher myogenin and MyoD levels compared to cells co-transfected with negative siRNA and AdC. GAPDH was used as an internal control. (C) Myotubes transfected as described above were treated with EdU and detected by immunofluorescence. Cells transfected with siRNA myogenin showed significantly high levels of EdU, compared to the cleaved cells co-transfected with myogenin siRNA and AdMyoD. Cells transfected with AdMyoD only or negative siRNA co-transfected with AdC showed no signs of cleavage.

**Figure 8:**
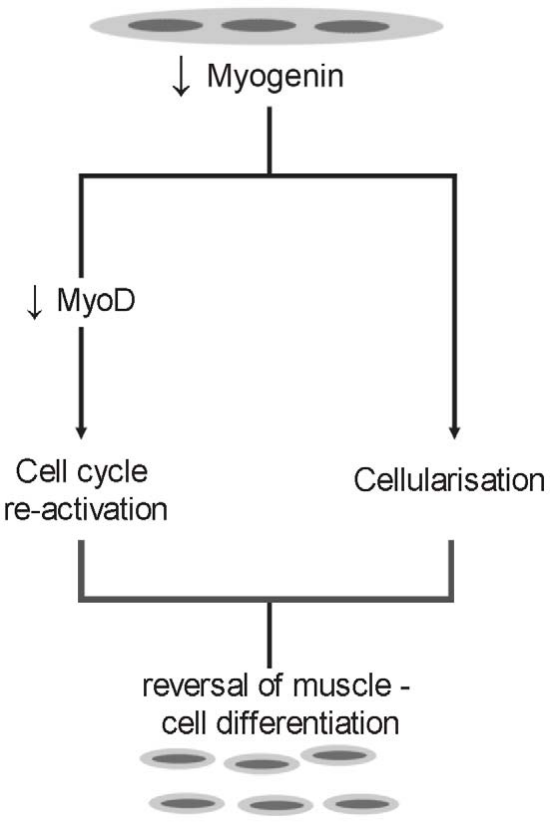
Hypothesis on myogenin-mediated reversal of muscle cell differentiation. Down-regulation of myogenin in terminally differentiated myotubes cleaves terminally differentiated cells into mononuclear cells which reenter cell cycle through down-regulation of MyoD. Based on the experiments performed in this work, it can be deduced that down-regulation of myogenin induces these two activities through separate pathways.

In order to characterize molecularly the effect of overexpression of MyoD in parallel with the down-regulation of myogenin, RNA analysis was carried out for molecules which are implicated in muscle cell differentiation and the cell cycle. Cells transfected with AdMyoD and myogenin siRNA had reduced endogenous myogenin RNA and protein levels and increased MyoD levels, similar to those detected in control transfected cells (fig. 7A, B). As a result of the repaired MyoD levels, no induction was observed in cyclin D1 and E2 levels (fig. 7A, B). Overexpression of MyoD successfully prevented cyclins from being induced from resting levels. This result, in combination with the cellularisation seen in cells transfected with AdMyoD and myogenin siRNA indicate that down-regulation of myogenin can cause cell cycle reentry and cleavage of myotubes into mononucleated cells possibly through two different pathways (fig. 8). Overexpression of MyoD only was also carried out in myotubes and showed increase in both MyoD and myogenin endogenous levels (fig. 7A, B). It is well possible that in the terminal differentiated state of myotubes, overexpression of MyoD induces myogenin expression, as it has been shown by others [37]. This does not happen in cells transfected also with siRNA myogenin, perhaps because reduction of endogenous levels of myogenin initiates reversal of differentiation first and does not allow induction of its expression by the overexpressed MyoD. Furthermore, cells which originated from the cleavage of myotubes transfected with myogenin siRNA, showed high EdU incorporation: almost all cleaved cells exhibited EdU incorporation (fig. 7C).

## Discussion

During this work, an attempt was made to investigate the role of the four MRFs in maintaining terminal differentiation in muscle cells. Down-regulation of myogenin in C2C12 mouse myotubes caused cleavage into mononucleated cells and entry into the cell cycle through down-regulation of MyoD. Results from this study show also that down-regulation of myogenin causes cleavage of myotubes through a mechanism which is independent of the cell cycle reentry.

MRFs and especially myogenin were shown to regulate fusion of myoblasts into multinucleated muscle cells. In myogenin-null mice, very few myoblasts are able to fuse even when these myoblasts are specified and position correctly in order to fuse [38], [39]. *In vivo*, targeted mutation in the myogenin gene caused the severe reduction of all skeletal muscle, showing its importance towards skeletal muscle development [33]. The fusion of myoblasts is one of the key steps of muscle cell differentiation. Myogenin, MyoD and Myf5 were shown to express in fusing myoblasts, with each having as targets a distinct subset on muscle specific genes at the on-set of fusion. Downregulation of these MRFs during fusion period showed that particularly myogenin significantly inhibited the fusion of myoblasts [40]. Our experiments show that down-regulation of myogenin in terminally differentiated myotubes induced cleavage of multinucleated myotubes into mononucleated cells, the opposite of fusion of myoblasts. This is an important finding with respect to the function of myogenin in myoblast fusion. Regarding the reversal of muscle cell differentiation, it is a novel finding that myogenin down-regulation initiates an unknown mechanism which results to the fragmentation of myotubes into mononucleated cells. It would be very important to indentify this pathway in future studies.

MyoD is one of the key transcription factors responsible for the differentiation of muscle cells. One of the main actions of MyoD is the withdrawal of myoblasts from the cell cycle, in order to initiate myogenesis [41]. As a result, MyoD is highly expressed in undifferentiated muscle cells and continues to be active during muscle cell differentiation [42]. Myf6 is highly expressed during the last stages of muscle cell differentiation and is only detectable in mature myofibers [42]. Molecular analysis performed in our study revealed that down-regulation of myogenin in terminally differentiated muscle cells reduced both MyoD and Myf6 levels. This seems to be supported from the fact that down-regulation of myogenin caused the reversal of differentiation. In contrast to these changes, Myf5 was upregulated. This is probably due to the compensation mechanism between MyoD and Myf5 [43]. Mice and muscle cells lacking MyoD are viable and fertile showing significantly elevated levels of Myf5 [44], while mice lacking both MyoD and Myf5 reveal a complete absence of skeletal muscle [35]. These findings suggest that either Myf5 or MyoD is required for the determination of skeletal myoblasts.

Furthermore, our experiments reveal re-activation of cell cycle as seen by DNA replication through EdU incorporation and the upregulation of cyclin D1 and cyclin E2. These results point to a mechanism whereby down-regulation of myogenin in terminally differentiated muscle cells induces a process of reversal of differentiation. As previously shown, down-regulation of myogenin in myotubes induces the reduction of MyoD levels. By transfecting these myotubes with an adenovirus expressing MyoD, endogenous MyoD levels were brought back to normal. These myotubes were able to cleave but re-entry to the cell cycle was not obtained. Cyclin D1 and cyclin E2 levels were similar to those expressed by normal differentiated myotubes. These results point to a mechanism whereby myogenin down-regulation is responsible for the cleavage of terminally differentiated myotubes into mononucleated cells in a separate way from the cell cycle re-entry. In support to these findings, mice expressing homozygous mutated myogenin gene show major reduction in skeletal muscle. In contrast, homozygous mutations of Myf5 or MyoD showed no effect on skeletal muscles [33], [45]. Moreover, myogenin is required for myoblast fusion and differentiation but not for commitment to the myogenic lineage [34].

Furthermore, cyclin D1 antagonizes the myogenic activity of MyoD [46], [47]. Cyclin E2 is highly activated during the G1 to S phase progression, with significant effects on cell cycle activity and DNA replication. This supports our findings, as cyclin E2 and DNA synthesis were found up-regulated. Interestingly, scientific evidence supports the hypothesis that cyclin E2 is in close proximity to cyclin D1. In the absence of cyclin D1, cyclin E2 was found to functionally replace cyclin D1 [48].

Our work with RNA interference has revealed that down-regulation of endogenous myogenin gene expression in muscle cells can lead to reversal of muscle cell differentiation and the creation of mononucleated cells. There is growing evidence from published findings from several groups that it is possible to reverse muscle cell differentiation in mammalian cells [7], [49], [50]. This report is based on manipulating endogenous levels in order to achieve reversal of differentiation.

## Materials and Methods

### Tissue culture

C2C12 mouse myoblasts (ECACC) were grown to confluency under 5% CO_2_ at 37°C in growth medium (GM), DMEM medium (Gibco) supplemented with 10% (v/v) fetal bovine serum (FBS) (Gibco), 2 mM glutamine (Gibco) and penicillin-streptomycin (100 µg/ml–100 U/ml) (Gibco). In order to differentiate, cells were then switched to differentiation medium (DM), DMEM supplemented with 2% horse serum (v/v) (Gibco), 2 mM glutamine and penicillin-streptomycin (100 µg/ml–100 U/ml) for 4 days. During the first two days of differentiation, cytosine β-D-arabinofuranoside (Ara-C) (Sigma) (4 µg/ml) was included in order to eliminate all possible undifferentiated myoblasts. Medium was then replaced with fresh DM medium without Ara-C. Ara-C purified myotubes contained more than 90% of nuclei. For siRNA transfections on differentiated myotubes 100 pmol of each siRNA (MyoD, myogenin, Myf6 and Myf5) (Invitrogen) in complex with 10 µl of Lipofectamine RNAi/MAX (Invitrogen) dissolved in Optimem solution (Gibco) were incubated in myotubes for 6 hours. Transfection mix was then substituted with fresh GM medium. The following day, cells were re-transfected with each siRNA and transfection mix was again substituted with fresh GM medium.

### Immunofluorescent studies

Cells were incubated with various antibodies after siRNA transfections and 0–3 days of growth medium incubation. Briefly, cells were fixed in 4% paraformaldehyde in PBS for 20 min, washed once with PBS and permeabilized with 0.2% Triton-X-100 in PBS for 20 min. Cells were blocked with 1% BSA in PBS for 10 min and then exposed to primary antibodies. Cells were tested for MHC (1/200; MY32, Sigma) and myogenin (1/200; Santa-Cruz) for 2 hours in a 37°C humidified incubator. The cells were washed three times with PBS and then treated with a secondary antibody (goat anti-mouse Texas Red; Jackson Immunoresearch) for 1 hour at room temperature. Cells were washed three times with PBS and observed with a Zeiss AxioVision observer using Texas Red filters. Fusion Index (FI) was calculated as the number of nuclei present in myotubes over the total number of nuclei present in the observed field. Data was selected from 10 different and randomly chosen microscopic fields.

### Cell cycle studies

Myotubes transfected with myogenin siRNA and incubated in GM for up to three days, were then supplemented with 5-ethynyl-2′-deoxyuridine (EdU) for 3 hours (following manufacturer's instructions) (Invitrogen). Cells were then fixed with 4% paraformaldehyde in PBS for 20 min. Cells were washed twice with 3% BSA in PBS, before and after permeabilization with 0.5% Triton-X-100 in PBS for 20 min at room temperature. Cells were then incubated for 30 min in a mixture containing Alexa fluor 647. Cells were then washed with PBS and observed under a fluorescence microscope.

### RNA analysis

Total RNA was extracted from transfected or untransfected myotubes (Perfect RNAEukaryotic Mini kit, Eppendorf) and then subjected to reverse transcription. For PCR, specific primers were used for the analysis of expression for the following molecules: MyoD F 5′-GCCCGCGCTCCAACTGCTCTGAT-3′, R 5′- CCTACGGTGGTGCGCCCTCTGC-3′. Myogenin F 5′-CATCCAGTACATTG AGCGCCTA-3′, R 5′-GAGCAAATGATCTCCTGGGTTG. Myf6 F 5′-ATG GTACCCTATCCCGTTGC-3′, R 5′-TAGCTGCTTTCCGACGATCT-3′. Myf5 F 5′-TGAAGGATGGACATGACGGACG-3′,R 5′-TTGTGTGCTCCGAAGGCTGCTA-3′. Cyclin D1 F 5′-GGCACCTGGATTGTTCTGCT-3′, R 5′-CAGCTTGC TAGGGAACTTGG-3′. Cyclin E2 F 5′-GGAACCACAGATGAGGTC-3′, R 5′-CG TAAGCAAACTCTTGGAG-3′. GAPDH F 5′-TCATCATCTCCGCCCCTTCT-3′, R 5′- GAGGGGCCATCCACAGTCTT-3′.

Experiments were repeated at least three times and gel bands were measured using the Scion Image software.

### Western blot

Cells were lysed using a protein lysis buffer including protease inhibitor. 40–60 µg of protein extracts were incubated with myogenin (1/200; BD), MyoD (1/300; Santa-Cruz), cyclin D1 (1/400; Abcam), cyclin E2 (1/200, Abcam) and GAPDH (1/2000; Santa-Cruz) primary antibodies followed by incubation with goat anti-mouse IgG or donkey anti-rabbit IgG secondary antibodies conjugated to horseradish peroxidase (Santa-Cruz).

## References

[1] Brockes JP, Kumar A (2005). Appendage regeneration in adult vertebrates and implications for regenerative medicine.. Science.

[2] Morrison JI, Loof S, He P, Simon A (2006). Salamander limb regeneration involves the activation of a multipotent skeletal muscle satellite cell population.. J Cell Biol.

[3] Brockes JP, Kumar A (2002). Plasticity and reprogramming of differentiated cells in amphibian regeneration.. Nat Rev Mol Cell Biol.

[4] Olson EN, Brennan TJ, Chakraborty T, Cheng TC, Cserjesi P (1991). Molecular control of myogenesis: antagonism between growth and differentiation.. Mol Cell Biochem.

[5] Mohun T (1992). Muscle differentiation.. Curr Opin Cell Biol.

[6] McGann CJ, Odelberg SJ, Keating MT (2001). Mammalian myotube dedifferentiation induced by newt regeneration extract.. Proc Natl Acad Sci U S A.

[7] Odelberg SJ, Kollhoff A, Keating MT (2000). Dedifferentiation of mammalian myotubes induced by msx1.. Cell.

[8] Hjiantoniou E, Anayasa M, Nicolaou P, Bantounas I, Saito M (2008). Twist induces reversal of myotube formation.. Differentiation.

[9] Meech R, Gomez M, Woolley C, Barro M, Hulin JA (2010). The homeobox transcription factor Barx2 regulates plasticity of young primary myofibers.. PLoS One.

[10] Duckmanton A, Kumar A, Chang YT, Brockes JP (2005). A single-cell analysis of myogenic dedifferentiation induced by small molecules.. Chem Biol.

[11] Rosania GR, Chang YT, Perez O, Sutherlin D, Dong H (2000). Myoseverin, a microtubule-binding molecule with novel cellular effects.. Nat Biotechnol.

[12] Jung DW, Williams DR (2011). Novel Chemically Defined Approach To Produce Multipotent Cells from Terminally Differentiated Tissue Syncytia.. ACS Chem Biol.

[13] Pajalunga D, Puggioni EM, Mazzola A, Leva V, Montecucco A (2010). DNA replication is intrinsically hindered in terminally differentiated myotubes.. PLoS One.

[14] Mu X, Peng H, Pan H, Huard J, Li Y (2011). Study of muscle cell dedifferentiation after skeletal muscle injury of mice with a Cre-Lox system.. PLoS One.

[15] Echeverri K, Tanaka EM (2002). Mechanisms of muscle dedifferentiation during regeneration.. Semin Cell Dev Biol.

[16] Buckingham M (2001). Skeletal muscle formation in vertebrates.. Curr Opin Genet Dev.

[17] Megeney LA, Rudnicki MA (1995). Determination versus differentiation and the MyoD family of transcription factors.. Biochem Cell Biol.

[18] de la Serna IL, Carlson KA, Imbalzano AN (2001). Mammalian SWI/SNF complexes promote MyoD-mediated muscle differentiation.. Nat Genet.

[19] de la Serna IL, Roy K, Carlson KA, Imbalzano AN (2001). MyoD can induce cell cycle arrest but not muscle differentiation in the presence of dominant negative SWI/SNF chromatin remodeling enzymes.. J Biol Chem.

[20] Olson EN (1992). Interplay between proliferation and differentiation within the myogenic lineage.. Dev Biol.

[21] Olson EN, Klein WH (1998). Muscle minus myoD.. Dev Biol.

[22] Molkentin JD, Olson EN (1996). Defining the regulatory networks for muscle development.. Curr Opin Genet Dev.

[23] Kitzmann M, Carnac G, Vandromme M, Primig M, Lamb NJ (1998). The muscle regulatory factors MyoD and myf-5 undergo distinct cell cycle-specific expression in muscle cells.. J Cell Biol.

[24] Halevy O, Novitch BG, Spicer DB, Skapek SX, Rhee J (1995). Correlation of terminal cell cycle arrest of skeletal muscle with induction of p21 by MyoD.. Science.

[25] Guo K, Wang J, Andres V, Smith RC, Walsh K (1995). MyoD-induced expression of p21 inhibits cyclin-dependent kinase activity upon myocyte terminal differentiation.. Mol Cell Biol.

[26] Cenciarelli C, De Santa F, Puri PL, Mattei E, Ricci L (1999). Critical role played by cyclin D3 in the MyoD-mediated arrest of cell cycle during myoblast differentiation.. Mol Cell Biol.

[27] Thorburn AM, Walton PA, Feramisco JR (1993). MyoD induced cell cycle arrest is associated with increased nuclear affinity of the Rb protein.. Mol Biol Cell.

[28] Camarda G, Siepi F, Pajalunga D, Bernardini C, Rossi R (2004). A pRb-independent mechanism preserves the postmitotic state in terminally differentiated skeletal muscle cells.. J Cell Biol.

[29] Blais A, Tsikitis M, Acosta-Alvear D, Sharan R, Kluger Y (2005). An initial blueprint for myogenic differentiation.. Genes Dev.

[30] Edmondson DG, Olson EN (1989). A gene with homology to the myc similarity region of MyoD1 is expressed during myogenesis and is sufficient to activate the muscle differentiation program.. Genes Dev.

[31] Rhodes SJ, Konieczny SF (1989). Identification of MRF4: a new member of the muscle regulatory factor gene family.. Genes Dev.

[32] Wright WE, Sassoon DA, Lin VK (1989). Myogenin, a factor regulating myogenesis, has a domain homologous to MyoD.. Cell.

[33] Hasty P, Bradley A, Morris JH, Edmondson DG, Venuti JM (1993). Muscle deficiency and neonatal death in mice with a targeted mutation in the myogenin gene.. Nature.

[34] Myer A, Olson EN, Klein WH (2001). MyoD cannot compensate for the absence of myogenin during skeletal muscle differentiation in murine embryonic stem cells.. Dev Biol.

[35] Rudnicki MA, Schnegelsberg PN, Stead RH, Braun T, Arnold HH (1993). MyoD or Myf-5 is required for the formation of skeletal muscle.. Cell.

[36] Masamha CP, Benbrook DM (2009). Cyclin D1 degradation is sufficient to induce G1 cell cycle arrest despite constitutive expression of cyclin E2 in ovarian cancer cells.. Cancer Res.

[37] Qin RF, Mao TQ, Gu XM, Hu KJ, Liu YP (2007). Regulation of skeletal muscle differentiation in fibroblasts by exogenous MyoD gene in vitro and in vivo.. Mol Cell Biochem.

[38] Myer A, Wagner DS, Vivian JL, Olson EN, Klein WH (1997). Wild-type myoblasts rescue the ability of myogenin-null myoblasts to fuse in vivo.. Dev Biol.

[39] Rawls A, Morris JH, Rudnicki M, Braun T, Arnold HH (1995). Myogenin's functions do not overlap with those of MyoD or Myf-5 during mouse embryogenesis.. Dev Biol.

[40] Dedieu S, Mazeres G, Cottin P, Brustis JJ (2002). Involvement of myogenic regulator factors during fusion in the cell line C2C12.. Int J Dev Biol.

[41] Crescenzi M, Fleming TP, Lassar AB, Weintraub H, Aaronson SA (1990). MyoD induces growth arrest independent of differentiation in normal and transformed cells.. Proc Natl Acad Sci U S A.

[42] Buckingham M (1994). Muscle differentiation. Which myogenic factors make muscle?. Curr Biol.

[43] Braun T, Rudnicki MA, Arnold HH, Jaenisch R (1992). Targeted inactivation of the muscle regulatory gene Myf-5 results in abnormal rib development and perinatal death.. Cell.

[44] Rudnicki MA, Braun T, Hinuma S, Jaenisch R (1992). Inactivation of MyoD in mice leads to up-regulation of the myogenic HLH gene Myf-5 and results in apparently normal muscle development.. Cell.

[45] Nabeshima Y, Hanaoka K, Hayasaka M, Esumi E, Li S (1993). Myogenin gene disruption results in perinatal lethality because of severe muscle defect.. Nature.

[46] Rao SS, Chu C, Kohtz DS (1994). Ectopic expression of cyclin D1 prevents activation of gene transcription by myogenic basic helix-loop-helix regulators.. Mol Cell Biol.

[47] Rao SS, Kohtz DS (1995). Positive and negative regulation of D-type cyclin expression in skeletal myoblasts by basic fibroblast growth factor and transforming growth factor beta. A role for cyclin D1 in control of myoblast differentiation.. J Biol Chem.

[48] Geng Y, Whoriskey W, Park MY, Bronson RT, Medema RH (1999). Rescue of cyclin D1 deficiency by knockin cyclin E.. Cell.

[49] Schneider JW, Gu W, Zhu L, Mahdavi V, Nadal-Ginard B (1994). Reversal of terminal differentiation mediated by p107 in Rb−/− muscle cells.. Science.

[50] Perez OD, Chang YT, Rosania G, Sutherlin D, Schultz PG (2002). Inhibition and reversal of myogenic differentiation by purine-based microtubule assembly inhibitors.. Chem Biol.

